# Tailored internet-delivered cognitive behavior therapy for depression in older adults: a randomized controlled trial

**DOI:** 10.1186/s12877-024-05597-8

**Published:** 2024-12-10

**Authors:** Lise Bergman Nordgren, Mikael Ludvigsson, Kristin Silfvernagel, Linnéa Törnhage, Lisa Sävås, Sophie Söderqvist, Sofia Dinnetz, Paulina Henrichsén, Johanna Larsson, Hanna Ström, Malin Lindh, Thomas Berger, Gerhard Andersson

**Affiliations:** 1 Region Örebro län and Department of medicine, Faculty of Medicine and Health, Örebro University, Örebro, Sweden; 2 Department of Psychiatry in Linköping, Department of Biomedical and Clinical Sciences, Linköping University, Linköping, Sweden; 3 Department of Acute Internal Medicine and Geriatrics in Linköping, Department of Health, Medicine and Caring Sciences, Linköping University, Linköping, Sweden; 4 Department of Behavioural Sciences and Learning, Linköping University, Linköping, Sweden; 5 Department of Clinical Psychology and Psychotherapy, University of Bern, Bern, Switzerland; 6 Department of Clinical Neuroscience, Karolinska Institute, Stockholm, Sweden

**Keywords:** eHealth, Internet, Cognitive function, Access, Mental health care

## Abstract

**Background:**

Depression is a common and serious problem in older adults, but few have access to psychological treatments. Internet-delivered Cognitive Behavioral Therapy (ICBT) has the potential to improve access and has been found to be effective in adults with depression. The aim of this study was to examine the effects of tailored ICBT for depression in older adults aged 65 years or older. We also investigated if cognitive flexibility could predict outcome.

**Methods:**

Following online recruitment from the community, included participants were randomly allocated to either ten weeks of clinician guided ICBT (*n* = 50) or to an active control group in the form of non-directive support (*n* = 51). Primary depression outcome was the Geriatric Depression Scale (GDS-15). Several secondary outcomes were used, such as the Beck Depression Inventory (BDI-II) and the Patient Health Questionnaire (PHQ-9).

**Results:**

Both treatment and active control groups significantly reduced their levels of depression, and the treatment group showed significantly greater improvement on the GDS-15 and BDI-II, but not on the PHQ-9. Between-group effect sizes as Cohen’s *d* were 0.78 (CI95% 0.36–1.20) on the GDS-15 and 0.53 (CI95% 0.11–0.94) on the BDI-II.

**Conclusions:**

Tailored ICBT is superior to an active control for older adults with depression. Between-group effects were smaller than in previous RCTs, most likely because of the use of an active control condition. Cognitive flexibility did not predict outcome. We conclude that ICBT can be used for older adults with depression, and thus increase access to psychotherapy for this group.

**Trial registration:**

This trial was retrospectively registered in clinicaltrials.gov (no. NCT05269524) the 8th of March 2022.

**Supplementary Information:**

The online version contains supplementary material available at 10.1186/s12877-024-05597-8.

## Background and objectives

Depression and subsyndromal depression in older adults are common and associated with poor quality of life, poor physical health, impaired activities in daily life, and a higher mortality [1, 2]. Among the different treatment alternatives, it has been argued that psychological treatment should be prioritized for older people because pharmacological treatments often involve side effects and interactions with other drugs and somatic diseases [3, 4]. Among different psychological treatments, Cognitive Behavioral Therapy (CBT), and especially the specific subtype Problem Solving Therapy, have good evidence for efficacy [5, 4]. Older people often prefer psychological treatment before pharmacological depression treatment, but even if psychological treatment is recommended as a first-line treatment, few older adults are offered such treatments [6, 7]. Reasons behind the low availability of psychological treatments for older people can include stigmatization when seeking help for mental illness, lack of transport facilities to be able to see a psychotherapist, and lack of confidence in treatment effect in therapist and client [8, 9].

To reduce the barriers of stigma and lack of transport facilities, internet-delivered CBT (ICBT) has been developed and tested for a range of conditions and target populations [10]. As the older adult can receive the treatment via the internet in their home environment, no transport is needed, and the stigma can be reduced in relation to the family, with access to treatment ensured without surrounding people even knowing about the mental illness or the associated psychotherapy. Therapist-supported ICBT has been found to provide effects comparable to those from face-to-face treatments for adults [11], and studies also suggest that ICBT can be effective for older adults [12, 13]. However, there are still few studies on older adults with depression. In a recent review, Xiang et al. only found three published RCTs investigating ICBT for depression for older adults [13]. In addition to the limited number of participants in these studies, all three studies were from Australia [14–16]. Another limitation has been that previous studies have used the same manual-based treatment package for all study participants, while there are reasons to believe that more individual adaptation can be beneficial both from a needs perspective and to increase motivation during treatment [17, 13]. In many clinical settings it is not possible to choose between different forms of psychological treatments, but based on individual case formulations, the components of ICBT can be tailored. Tailoring ICBT based on a selection of treatment modules has been tested in several studies including one on older adults with anxiety disorders, but has not yet been studied for depression in older adults [18, 19]. The main purpose of the present study was to adapt and test the effects of tailored therapist guided ICBT for older adults with depression in a controlled trial comparing against an active attention control condition.

One barrier against providing psychological treatments for older adults is the lack of confidence in treatment effects among both therapists and clients [8, 9]. One aspect that may contribute to such a lack of confidence is the notion that older adults would not be able to assimilate treatment due to cognitive limitations [8, 20]. This may contribute to older adults not being offered psychological treatments in health care (i.e., age discrimination). Studies on psychological treatments for depression in older adults with confirmed cognitive impairment have shown heterogeneous findings. For example, it has been reported that CBT has relatively good effects in the presence of cognitive deficits, or even in dementia [21, 20]. Previous studies of ICBT for depression in older adults have not evaluated cognitive functions in detail, although cognitive impairment has been used as an exclusion criterion [13].

In sum, as perceived cognitive function might act as a barrier against older adults’ access to ICBT, a second purpose of this study was to investigate whether cognitive function could predict treatment outcome. We hypothesized that ICBT for depression among older adults would have a better effect than active control, and that lower cognitive function would predict smaller treatment gains on depressive symptoms.

## Methods

### Setting and participants

The study was carried out in Sweden during 2016 and 2017. In general, internet usage among older adults in Sweden has been higher than among older adults in many other countries [22]. On the other hand, psychological treatment in Sweden is less commonly provided for older adults and is generally uncommon, just as in many other countries [23, 7]. Information about the study was advertised both on public sites on the web (including social media) and via flyers in other settings in Sweden (e.g., clinics). The screening procedure and the treatment were delivered via Iterapi [24], an internet platform built to be secure, efficient and easy to adapt to the tailored format. The information gathering, treatment, and communication were accessed through logging on using an individual study code and double authentication.

Applicants showed their interest by completing the online screening procedure consisting of demographic questions, questions on somatic disorders, previous or ongoing psychological or pharmacological treatment, and measures of depression, anxiety, quality of life, self-reported cognitive function, and alcohol use. Open questions about somatic disorders were asked to identify obstacles for participation according to exclusion criteria. Following this initial screening procedure, eligible applicants were contacted for a structured telephone-administered diagnostic interview using the Mini International Neuropsychiatric Interview (MINI) 7.0 [25]. The MINI was conducted by the therapists (presented below) after an introductory training. If included, participants were given a link to an online test of cognitive flexibility.

**Fig. 1:**
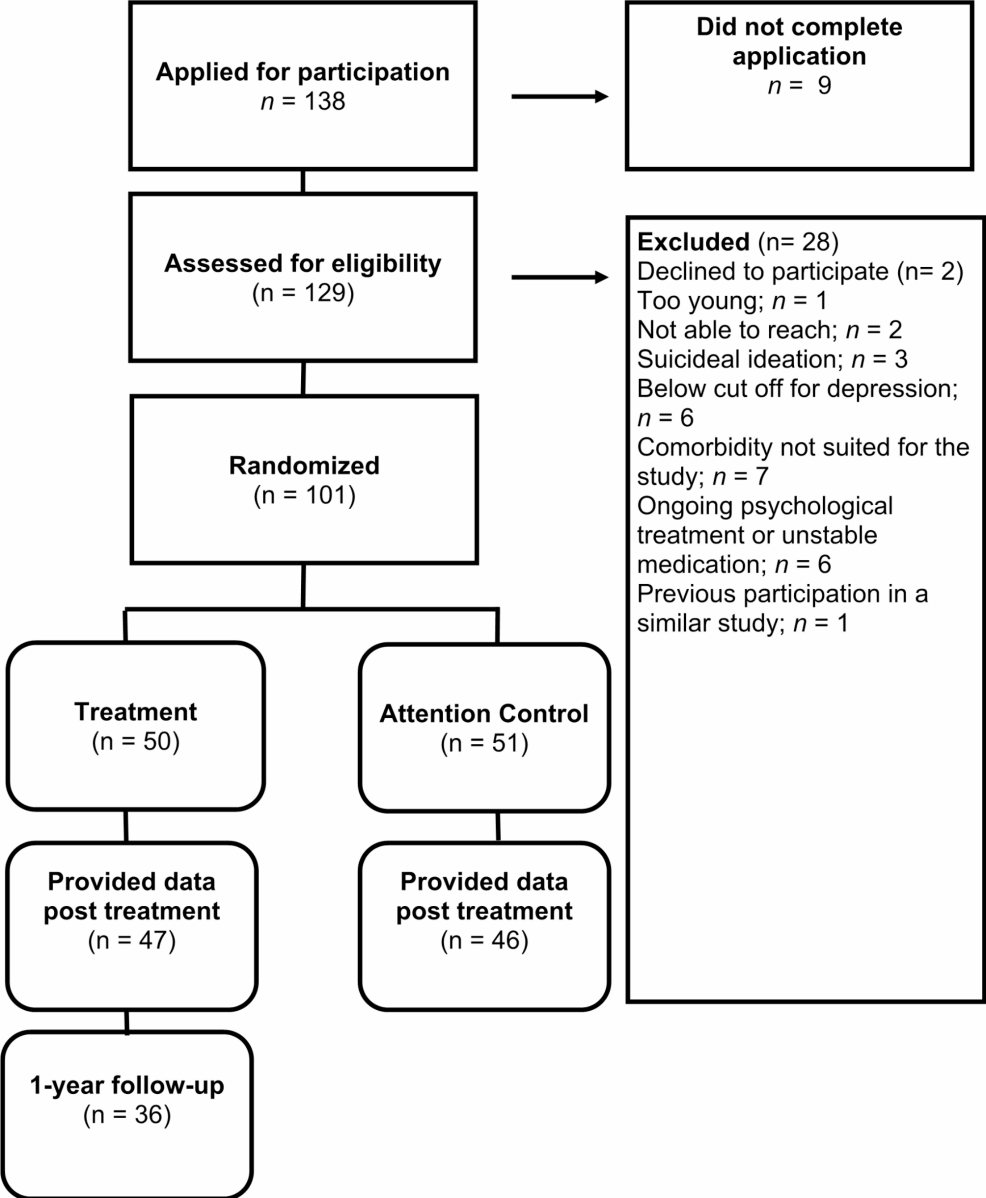
Flowchart of study participants, reasons for exclusion, point of random assignment

Recruitment was conducted on two separate occasions: time one in spring 2016 and time two in spring 2017. One hundred and thirty-eight people applied for participation and a total of 101 participants, aged between 65 and 88 years, were included after completion of the screening procedures. Eligible participants’ data were discussed during case management meetings with the researchers, and decisions about inclusion or exclusion were made based on formal criteria (Fig. 1). Inclusion criteria were age 65 years or older, presence of depression or subsyndromal depression, living in Sweden, and access to a computer and to the internet on a regular basis. Predefined exclusion criteria were substantial suicidal ideation, substantial alcohol abuse, other ongoing psychological treatment, unstable psychopharmacological medication, and severe psychiatric or somatic comorbidity that could interfere with the treatment provided in the study. Excluded persons were referred to appropriate care units depending on the individual type of problem. No exclusion was made based on somatic comorbidity, and exclusion for psychiatric reasons were based on symptom severity and functional impairments rather than diagnostic category.

Randomization to either treatment or control condition (1:1) was conducted with a random number generator (www.random.org) by a person who was not part of the study team. Descriptive statistics of the participants in both conditions are presented in Table 1. The study was approved by the local regional ethical board in Linköping, Sweden (Registration no. 2015/310 − 31), and written informed consent was collected via surface mail at inclusion. The study is reported according to CONSORT 2010 statement and registered in clinicaltrials.gov (no. NCT05269524, the 08/03/2022). The registration was made during the data analysis, as we discovered that it had not been done before by mistake.

**Table 1:** Participant characteristics of the treatment and control groups, overall *n* = 101

Variable		Treatment group *n* = 50	Control group *n* = 51	Overall *n* = 101	Independent t-value; *p*-value	χ2; *p*-value
Age, years mean (SD)		71.5 (4.7)	72.4 (4.2)	71.9 (4.4)	0.99; 0.325	
Female gender n(%)		35 (70.0)	36 (70.6)	71 (70.3)		< 0.01; 1.000
Living alone, n(%)		19 (38.0)	23 (45.1)	42 (41.6)		0.52; 0.546
Have no children, n(%)		16 (32.0)	16 (31.4)	32 (31.7)		0.01; 1.000
Educational level: tertiary school, n(%)		31 (62.0)	33 (64.7)	64 (63.4)		0.08; 0.838
Prior psychological treatment, n(%)		28 (56.0)	23 (45.1)	51 (50.5)		1.20; 0.322
Never psychopharmacological treatment, n(%)		20 (40.0)	29 (56.9)	49 (48.5)		2.87; 0.112
Any somatic disorder, n(%)		44 (88.0)	32 (62.7)	76 (75.2)		8.65; 0.005**
Any somatic disorder except hypertension, n(%)		34 (68.0)	28 (54.9)	62 (61.4)		1.83; 0.221
Psychiatric disorders						
	Anxiety disorder: GAD, Social anxiety disorder, panic disorder, Agoraphobia, OCD	18 (36.0)	20 (39.2)	38 (37.6)		0.11; 0.739
	Post-traumatic stress	2 (4.0)	1 (2.0)	3 (3.0)		0.36; 0.546
	Alcohol Use Disorder	3 (6.0)	2 (3.9)	5 (5.0)		0.23; 0.630
	Eating Disorder	2 (4.0)	1 (2.0)	3 (3.0)		0.36; 0.546
	Major depressive disorder	35 (70.0)	38 (74.5)	73 (72.3)		0.26; 0.613
	Subsyndromal depression	15 (30.0)	13 (25.5)	28 (27.7)		0.26; 0.613
Baseline measures						
	GDS-15, mean (SD)	8.8 (2.9)	9.0 (3.0)	8.9 (2.9)	0.37; 0.709	
	BDI-II, mean (SD)	23.5 (7.4)	24.9 (8.1)	24.2 (7.7)	0.92; 0.358	
	PHQ-9, mean (SD)	12.4 (4.3)	14.2 (4.0)	13.3 (4.2)	2.17; 0.032*	
	CFQ, mean (SD)	42.3 (14.3)	43.0 (11.8)	42.7 (13.0)	0.27; 0.790	
	WCST, mean (SD)	17.7 (7.8)	16.2 (6.4)	17.0 (7.1)	1.05; 0.298	

Note: GAD = Generalized Anxiety Disorder; OCD = Obsessive Compulsive Disorder; GDS = Geriatric Depression Scale; BDI-II = Beck Depression Inventory; PHQ = Patient Health Questionnaire; CFQ = Cognitive Failures Questionnaire; WCST = Wisconsin Card Sorting Test. *=*p* < 0.05; **=*p* < 0.01

### Design and procedure

Tailored ICBT, adapted for older adults through scheduled text-based therapist guidance (no automatic messages were sent), was compared against an active control condition consisting of a non-directive supportive contact on a weekly basis. Participants were people aged 65 years or older suffering from depression or subsyndromal depression. Both self-reported assessment and a computerized test of cognitive function were administered before treatment to investigate if either of these would predict treatment effects on depressive symptoms.

#### Treatment

The treatment period lasted 10 weeks and included, at the most, 10 modules per participant (range 6–10 modules that were selected from a total of 12 modules). All modules were based on CBT and had previously been developed and tested for adults with specific conditions (e.g., depression, panic disorder). They were modified to fit together in a tailored format. For example, words relating to specific conditions were removed, texts were made more legible, and audio files and videos were included. The content and form were also adapted to the context of older people (e.g., case descriptions including retirement and the loss of a partner in old age).

For a brief description of the treatment modules, see additional file 1. Five of the modules were offered as a standard (introduction, behavioral activation part one and two, acceptance, and relapse prevention), while the treatment was tailored by adding any of the following seven modules: sleep, anxiety, emotion regulation, loneliness, life review, pain, and an applied relaxation module. The tailoring of the treatment was based on the results of the screening measures, therapists’ and principal investigator assessments of the participant’s needs expressed in the telephone interview, and the participant’s own preferences. Each module consisted of a presentation of the targeted topic, a rationale for the exercises linked to the presented topic, and homework assignments for the participant to complete and report to their therapist for feedback on a weekly basis. The therapists gave feedback on the assignments weekly through text messages via the platform. Additional support was given on demand of the participants within 24 h. When the participant seemed to have mastered the key aspects of the module, the next prescribed module was made accessible. The treatment period ended after ten weeks no matter how many modules the participant had used.

All therapists (*n* = 8) were master students in clinical psychology, were in their final term of a five-year clinical psychology program. They had all completed their supervised clinical training and were also trained in delivering ICBT before the start of the study. Supervision was provided on a regular basis by a clinical psychologist and psychotherapist with experience of both regular clinical work with patients suffering from depression and internet-delivered treatments.

#### Active control condition: non-directive support

Participants in the control condition did not follow any structured manual but were asked questions about their well-being weekly by an identified therapist via the treatment platform (e.g., name and picture). Therapists were instructed according to a short manual to respond in a supportive and non-judgmental manner, but not to give any advice or recommendation related to the treatment modules (except for possible urgent matters in case of deterioration and technical problems).

### Measures

#### Primary outcome measures

Measurements were collected for all participants pre-treatment and post-treatment, while only the treatment group was measured at the one-year follow-up. The active control group was not tested at 12 months follow-up as they were offered active treatment after the study treatment period. All measures were administered online. The primary outcome was the 15-item Geriatric Depression Scale (GDS-15) [26]. It contains 15 questions with yes/no-answers with one point for each answer indicating depression. On the GDS-15 scores range between 0 and 15, and five or less are considered within normal range. The scale is widely used and has a high internal consistency.

#### Secondary outcome measures

We used two other measures of depressive symptoms. The Beck Depression Inventory-II (BDI-II) [27, 28], which contains of 23 ordinal questions about depression and a maximum score 63. A higher score indicates more depressive symptoms. We also used the Patient Health Questionnaire-9 (PHQ-9) [29, 30]. The PHQ-9 contains 9 ordinal items about depressive symptoms. The total score ranges from 0 to 27, with higher scores indicating more depressive symptoms. Subsyndromal depression was, in this study, defined as depressive symptomatology below the cutoff for major depression according to the MINI-interview, but with a minimum of (a) at least three depressive symptoms according to the MINI-interview, (b) a score of at least five on the PHQ-9, and (c) a significant suffering.

Anxiety symptoms were measured using the seven-item Generalized Anxiety Disorder scale (GAD-7) [31] and the Beck Anxiety Inventory (BAI) [32]. GAD-7 contains 7 ordinal questions with a total score ranging from 0 to 21, and higher scores indicating more anxiety. The BAI contains 21 ordinal items of frequency, with total score range between 0 and 63, and higher scores indicating more anxiety. Quality of life was measured using the Quality of Life Inventory (QOLI) [33]. The QOLI contains 16 Likert items, and total score ranges from − 6 to 6 with higher scores indicating a better quality of life.

#### Cognitive measures

Cognitive function was measured using the 25-item Cognitive Failures Questionnaire (CFQ) [34] for self-assessed overall cognitive ability, and the number of perseverative errors on the Wisconsin Card Sorting Test-64 (WCST) [35] as a measure of cognitive flexibility. The CFQ contains 25 ordinal questions with a total score ranging from 0 to 100 points, and higher scores indicating more perceived cognitive difficulties. The WCST used in this study was an in-house adapted online version of the standard, single-deck 64 response cards version of the WCST [35], which we have used in previous studies [36, 19]. The number of perseverative errors were counted, with higher counts indicating less flexible thinking [37]. The WCST was used only at pre-treatment, while all other measures were administered at three time points: pre-treatment, post-treatment and one-year follow-up.

### Statistical analyses

All statistical analyzes were performed using IBM SPSS Statistics 24. A *p*-value < 0.05 was considered statistically significant. The a priori power calculated with G*Power, gave that 84 participants, 42 in each condition, would be sufficient to discover large between-group effect sizes between the treatment and control conditions at post-treatment assessment [38]. We also considered dropout and therefore planned for an overinclusion of participants with an expected dropout rate of at least 20%. We based the expectation of a large effect (Cohen’s *d* 0.80) on previous ICBT studies on depression in older adults [13]. Although we had an active control condition, in terms of effect we regarded this as closer to a passive control group compared to a hypothetical treatment condition that would require the same amount of work as a CBT condition. Post hoc tests revealed that our final sample of 101 patients, and our moderate effect sizes, rendered a power of 70% using the initial alpha of 0.05. Analysis of Covariance (ANCOVA) was used with pre-treatment scores as covariates for all the self-report measures to investigate the treatment effects [39]. Assumptions for ANCOVA were checked before (e.g., homogeneity of variances) and found to be robust. Linear regression analyses were performed to evaluate CFQ and WCST as possible predictors of treatment outcome. Effect sizes (Cohens *d*) were calculated to investigate within-group and between-group effect sizes. In the latter case we used model derived means from the ANCOVA models and observed SD’s. As the response rate were high with very few missing cases, the analyses were run based on complete cases [40], assuming that the few missing cases were Missing completely at random (MCAR).

Measures were collected on an intention-to-treat principle regardless of how many treatment modules participants had completed.

## Results

### Dropout and adherence

Only 8% (8/101) dropped out at post-treatment assessment (see Fig. 1). At one-year follow-up, 11 additional participants dropped out in the treatment group (23%; 11/47). On average 8.75 (SD = 0.74) modules were prescribed and the average number of completed modules was 6.63 (SD = 2.99; range 0–10), which means that 75.7% of the modules prescribed were completed. No adverse events were reported. On average, therapist time per participant and week was 16.8 (SD = 13.0) minutes for the treatment group and 5.5 (SD = 5.8) minutes for the control group.

### Baseline measures

T-tests and Chi-square tests showed a lower proportion of reported somatic disorders in the control group (32/51; 62.7%) compared to the ICBT group (44/50; 88.0%) (Table 1). In addition, PHQ-9 showed more depressive symptoms in the control group than in the ICBT group (M = 14.2, SD = 4.2 vs. M = 12.5, SD = 4.4; *p* = 0.03) at baseline.

### Treatment outcomes post-treatment

**Fig. 2:**
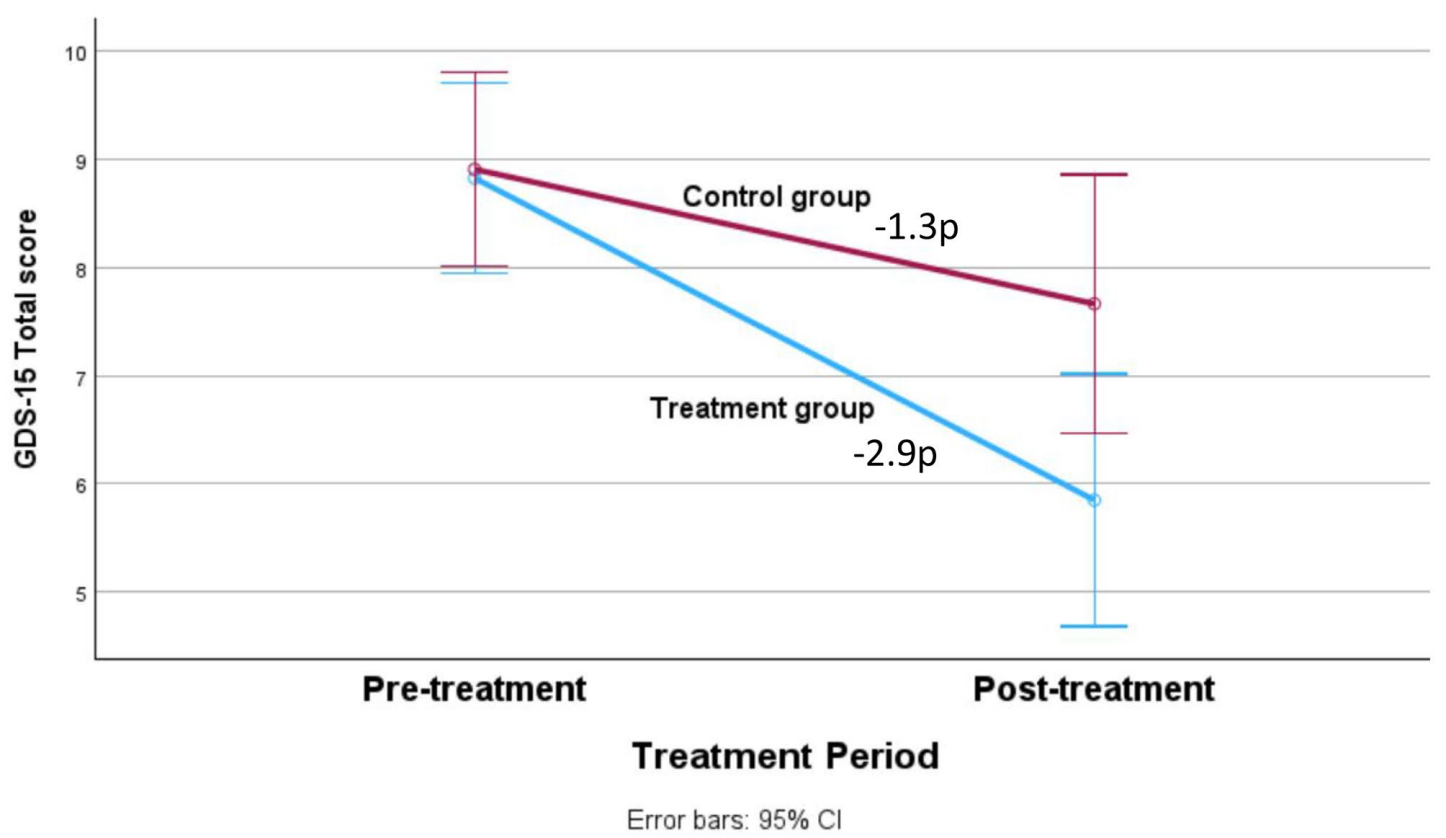
Mean changes of depressive symptoms measured with GDS-15 pre and post treatment

**Fig. 3:**
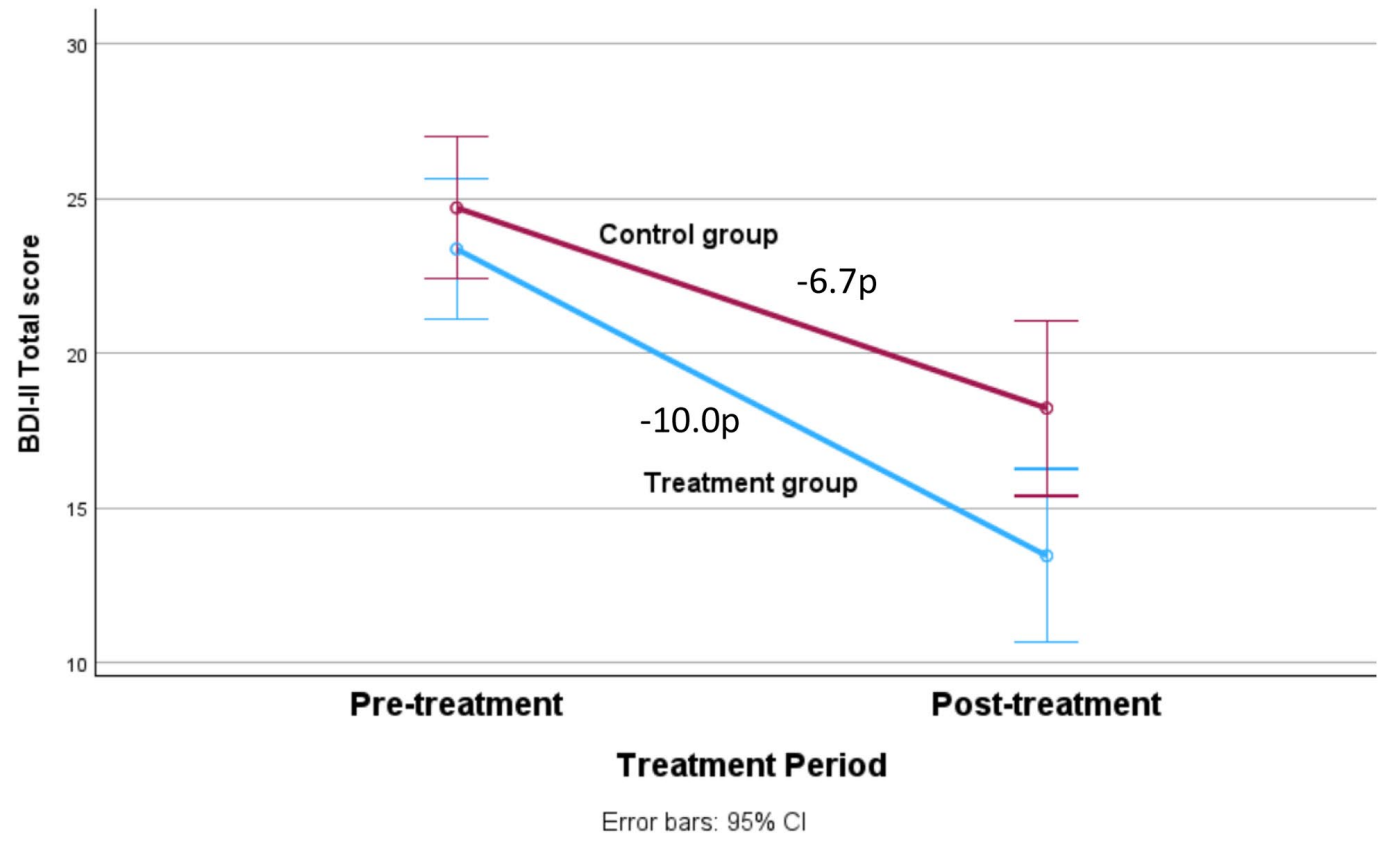
Mean changes of depressive symptoms measured with BDI-II pre and post treatment

Results and effect sizes are reported in Table 2, and the depression results are presented graphically in Figs. 2 and 3. Both the treatment group and the control group improved significantly on the primary outcome measure and on some secondary outcomes. The treatment group benefited more than the control group on the primary outcome measure GDS-15 (*F*(1, 90) = 7.28; *p* = 0.008; Fig. 2). This was also observed on the secondary outcome BDI-II (*F*(1, 90) = 5.34; *p* = 0.023; Fig. 3), but not on the PHQ-9 (*F*(1, 90) = 0.60; *p* = 0.441). The between-group effect sizes were moderate for the depression measures with significant results (GDS-15: *d* = 0.78; BDI-II: *d* = 0.53). For the other secondary measures, the treatment group benefited more than the control group on the QOLI (*F*(1, 90) = 8.04, *p* = 0.006) and the GAD-7 (*F*(1, 90) = 5.98, *p* = 0.016), but not on the BAI (*F*(1, 90) = 2.68, *p* = 0.105).

**Table 2:** Results on outcome measures: means, standard deviations, t-values, ancova results and effect sizes (Cohen’s d)

Measure		Pre Mean (SD)	Post Mean (SD)	Within-group paired t-value; *p*-value	Within-group Cohen’s d (95%CI)	ANCOVA F (df = 1,90); *p*-value	Between-group Cohen’s d (95%CI) ^a^
GDS-15							
	Treatment	8.8 (2.9)	5.9 (3.8)	6.36; <0.001	0.93 (0.58–1.27)	7.28; 0.008	0.78 (0.36–1.20)
	Control	9.0 (3.0)	7.7 (4.2)	2.78; < 0.008	0.41 (0.11–0.72)		
BDI-II							
	Treatment	23.5 (7.4)	13.5 (9.7)	7.15; <0.001	1.04 (0.68–1.40)	5.34; 0.023	0.53 (0.11–0.94)
	Control	24.9 (8.1)	18.2 (9.6)	6.65; <0.001	0.98 (0.62–1.33)		
PHQ-9							
	Treatment	12.4 (4.3)	6.6 (4.3)	8.12; <0.001	1.18 (0.81–1.55)	0.60; 0.441	0.25 (-0.15-0.65)
	Control	14.2 (4.0)	8.1 (4.9)	9.29; <0.001	1.37 (0.96–1.77)		
BAI							
	Treatment	15.0 (9.2)	10.0 (7.5)	6.05; <0.001	0.88 (0.54–1.22)	2.68; 0.105	0.13 (-0.27-0.54)
	Control	15.4 (9.2)	11.4 (7.9)	3.49; 0.001	0.51 (0.20–0.82)		
GAD-7							
	Treatment	7.5 (4.6)	3.6 (2.9)	5.73; <0.001	0.84 (0.50–1.16)	5.98; 0.016	0.50 (0.08–0.91)
	Control	7.9 (4.6)	5.5 (4.1)	4.46; <0.001	0.66 (0.34–0.97)		
QOLI							
	Treatment	0.4 (1.8)	1.2 (1.7)	5.11; <0.001	0.75 (0.42–1.07)	8.04; 0.006	1.00 (0.04–1.43)
	Control	0.6 (1.5)	0.7 (2.0)	0.33; 0.741	0.05 (-0.24-0.34)		
CFQ^b^							
	Treatment	42.3 (14.3)	36.6 (12.0)	4.28; <0.001	0.62 (0.31–0.93)	6.52; 0.012	0.38 (0.05–0.76)
	Control	43.0 (11.9)	41.0 (11.4)	2.08; 0.043	0.31 (0.01–0.60)		

Notes: GDS = Geriatric Depression Scale; BDI-II = Beck Depression Inventory; PHQ = Patient Health Questionnaire; BAI = Beck Anxiety Index; GAD = Generalized Anxiety Disorder; QOLI = Quality of Life Inventory; CFQ = Cognitive Failures Questionnaire. ^a^= Cohen’s *d* effect size based on estimated means from the ANCOVA model and observed SDs at baseline. ^b^= CFQ was not predefined as an outcome measure but is included in Table 2 as this measure changed in a similar way as the predefined outcome measures

### Cognitive function as a possible predictor of treatment outcome

Mean scores on the CFQ are presented in Table 2. The mean number of perseverative errors on the WCST was 15.32 (SD = 5.00). There was no significant correlation between cognitive flexibility and self-reported overall cognitive ability (treatment group *r* = 0.04, *p* = 0.70, control group; *r* = 0.05, *p* = 0.66). Neither cognitive flexibility nor self-assessed overall cognitive ability predicted any of the depression outcomes (based on change scores; Table 3). At post-treatment, assessment participants rated their overall cognitive ability significantly higher than at pre-treatment, and the ANCOVA showed a significant difference between groups (*F (*1, 90) = 6.52, *p* = 0.012).

**Table 3:** Baseline cognitive measures and their ability to predict depression outcome measured as change pre-post treatment, linear regression models, *n* = 101

	Predictor WCST pre treatment		Predictor CFQ pre Treatment	
Outcome variable	Univariate, unstandardized B (CI); p	Multivariate, Adjusted^a^ B (CI); p	Univariate, unstandardized B (CI); p	Multivariate, Adjusted^a^ B (CI); p
GDS-15 change pre-post	0.01 (-0.08-0.11): 0.786	0.01 (-0.08-0.10); 0.837	-0.01 (-0.06-0.04); 0.659	-0.01 (-0.06-0.04); 0.759
BDI-II change pre-post	0.23 (0.00-0.46); 0.051	0.22 (-0.01-0.45); 0.057	-0.11 (-0.24-0.02); 0.105	-0.11 (-0.24-0.02); 0.092
PHQ-9 change pre-post	-0.06 (-0.19-0.08); 0.419	-0.06 (-0.20-0.08); 0.383	-0.02 (-0.09-0.06); 0.609	-0.01 (-0.09-0.06-); 0.745
BAI change pre-post	0.07 (-0.12-0.26); 0.456	0.07 (-0.12-0.26); 0.477	-0.09 (-0.19-0.01); 0.086	-0.09 (-0.19-0.02); 0.108
GAD-7 change pre-post	-0.03 (-0.14-0.09); 0.627	-0.03 (-0.15-0.09); 0.611	-0.03 (-0.09-0.03); 0.313	-0.03 (-0.10-0.03); 0.289
QOLI change pre-post	-0.02 (-0.06-0.02); 0.303	-0.02 (-0.06-0.02); 0.396	0.00 (-0.02-0.02); 0.826	0.00 (-0.02-0.02); 0.920

Note: WCST = Wisconsin Card Sorting Test; CFQ = Cognitive Failures Questionnaire; GDS = Geriatric Depression Scale; BDI-II = Beck Depression Inventory; PHQ = Patient Health Questionnaire; BAI = Beck Anxiety Index; GAD = Generalized Anxiety Disorder; QOLI = Quality of Life Inventory

^a^ =Adjusted for the variables of age, gender and educational level

### One-year follow-up

As part of the design, a one-year follow-up was included for the treatment group (see additional file 2). The structured diagnostic interview revealed that the treatment group maintained their improvements, and at follow-up only 14% still fulfilled a diagnosis of depression, compared to 68% at baseline and 33% post-treatment. In terms of follow-up scores, the means for the GDS-15: 5.1 (SD = 3.7), BDI-II: 12.8 (SD = 8.3), PHQ-9: 6.9 (SD = 4.7), the BAI: 9.9 (SD = 7.1), the GAD-7: 4.3 (SD = 4.8), and QOLI: 1.2 (SD = 1.6), were all significantly lower than at pre-treatment. This indicates that treatment effects were maintained one year after treatment, although these results were based on the participants that had completed the one-year follow-up.

## Discussion and implications

### Main findings

The main purpose of the present study was to test the effects of tailored ICBT for older adults with depression, compared against an active control condition. The second purpose was to investigate if cognitive ability would predict treatment outcome. Results showed that the treatment group improved with reduced depression symptoms. However, given the use of an active control group we found improvements also in the control group. The treatment group improved more than the control group on the primary outcome, GDS-15 and on the secondary outcome BDI-II, but effect sizes were only moderate, and there was no significant difference on the PHQ-9. We also found improvements on the secondary outcomes QOLI and GAD-7, but not on the BAI for the treatment group compared to the control group. Long-term follow-up suggested that the effects were maintained, but at this stage the dropout rate had increased from the low initial rate of only 8%. We used two measures of cognitive function, and on the self-reported CFQ we found a significant treatment effect we had not expected with a small effect size in favor of the treatment. Neither self-reported cognitive failures (CFQ) nor cognitive flexibility (WCST) at pre-treatment did predict outcome, a finding that was against our hypothesis and contrary to previous results in older adults with anxiety disorders [19].

### Tailored ICBT for depression among older adults

Within-group measurements showed that depression was significantly reduced, with large effect sizes (Cohen’s *d* ranging between 0.93 and 1.18), indicating that tailored ICBT is an effective treatment for depression in older adults. However, as we used an active control condition, we also noted significant improvements in depression in the control group (Cohen’s d range 0.41–1.37), which reduced the between-group effects in line with expectations. There is a large body of literature suggesting that waitlist control groups can improve to some extent in iCBT studies [41]. The within-group effects were comparable or slightly smaller than in most of the other studies included in the review article reported by Xiang et al. [13], while the between-group effects were clearly smaller than in the only two RCTs reporting such analyses [14, 15], even if these studies used waitlist control and treatment as usual, respectively. It is well known that the type of control condition generally affects treatment outcomes in RCTs, and that active control can have powerful non-specific treatment effects. For example, participants, in receiving active control condition, can feel validated and possibly strengthened psychologically in a beneficial way [42].

One important difference between this study and previous studies of ICBT for depression among older adults is that we used tailored ICBT instead of a standardized transdiagnostic or depression-specific treatment. We believe that motivation, and indirectly treatment effects, can increase if the treatment is individualized in terms of components of a specific psychotherapy. This can be of particular relevance when implementing ICBT in a wider population. However, our study did not compare tailored versus a standardized treatment. Based on previous studies tailored ICBT appears to be a promising treatment option for older adults with mental health problems [18, 19]. Therapist time per participant and week was low compared to time general requirements in face-to-face therapy, which indicates that ICBT has advantages from a health economic perspective.

### Relevance of cognitive functions in ICBT in depression among older adults

Cognitive flexibility as measured by the WCST perseverative errors did not predict depression outcome measures in this study, which was contradictory to our hypothesis and earlier study [19]. This should not be interpreted as executive functions not being important. The finding might well be explained by a sample of participants with high executive functions or that the ICBT treatment format might be easier than regular psychotherapy when the client can work at his/her own pace, take pauses, and repeat parts of the treatment. Moreover, our careful individual adaptation (e.g., through simplified form and content of written material) of ICBT could compensate for certain executive disabilities in older adults with depression. In general, cognitive impairments can possibly be compensated by individualizing ICBT, and thus, cognitive impairment does not have to be an obstacle to offering ICBT to older adults with depression. This is supported by previous studies that indicated, for example, that older adults with depression and cognitive impairment or dementia may in fact benefit from CBT [21, 20]. However, we hesitate to draw any firm conclusions, even if the findings are in line with what we have seen for adults [36]. In addition to cognitive flexibility, there are other components of executive functions that could predict treatment outcomes or correspond to an association, if present.

Self-assessed overall cognitive ability also did not predict treatment outcomes in our study, but on the other hand, the scores on the CFQ changed significantly during the study, corresponding to a perceived improvement in cognitive function. It is previously known that cognitive functions vary with the degree of depression, and that they are recovered when a depression goes into remission [43]. Subjective estimation of overall cognitive function corresponding to CFQ is probably influenced by the negative attitudes of depressed people. Therefore, it is not surprising that the values of self-rated cognitive ability increased during the study as a possible effect of a reduced level of depression. One interpretation would be that subjectively estimated cognitive function could in principle be considered more as a marker of depression than as a marker for cognitive function in this sample. Such an interpretation is substantiated by previous studies [44]. In effect, the CFQ proved less relevant to evaluate the study’s research question of whether cognitive ability can predict treatment outcomes of depressive symptoms, although the instrument in general has relevance to other issues, such as memory failures, in everyday life.

### Practical implications and future directions

Although psychological treatment has been recommended as a first-line treatment for depression in older people [4], few older adults are offered this treatment option. ICBT has the potential to promote access to psychological treatment, for example by preventing the barriers of transport and stigma. However, empirical support is still limited when it comes to effects in older adults with depression. To further investigate the effects of ICBT on depression in older people, more studies are needed. In this study, we personalized the contents by tailoring the selection of treatment modules, as well as adapting the content and form to the unique needs of the older participants, but it is possible that such adaptations can be made even more effectively in future studies, with increased treatment motivation and greater treatment effects as possible consequences. The degree of cognitive flexibility did not predict the treatment outcome in this study, possibly indicating that many older people can assimilate ICBT treatment regardless of their ability for cognitive flexibility. Obviously, more severely cognitively impaired people would not enter a trial, and the role of cognitive impairment might be better studied in a clinical series of patients when the treatment is delivered in regular care. Moreover, future studies could focus on ways to further adapt ICBT in relation to cognitive functions to optimize treatment effects. It would also be useful to study other cognitive domains, such as, for example, episodic memory, visuospatial ability, and verbal fluency.

### Limitations and strengths of the study

This study has several strengths. Among the strengths were reaching a population that is rarely offered psychotherapy, the use of an active control condition, the very low initial dropout rate, and that we studied cognitive function using a performance test. However, there are also limitations that need to be considered. First, given the active control condition, this study had slightly limited statistical power, with relatively wide confidence intervals regarding effect sizes. We also acknowledge that a more active control group would have led to smaller effects and limited statistical power. Second, participants were recruited from the general public via advertisements, which entails a risk of sampling bias when compared to regular clinic patients. For example, it is possible that older adults with cognitive decline or less experienced internet users were less likely to apply for participating in the trial. On the other hand, it has been argued that samples recruited in clinics are also biased and that recruitment from the general public can at least be representative of this group [45]. Third, in line with many previous ICBT studies, participants did not complete all the assigned treatment modules, but the average number of completed modules was still high (75.7%) in relation to other studies [46]. On a related note, we had very few dropouts from assessment at posttreatment (8%), and it would have been preferable if not realistic to have no dropouts at all. Fourth, we lack follow-up data for the control group as these participants were offered active treatment after the ten weeks of treatment period. Fifth, we did not measure any specific placebo-relevant factors, such as treatment engagement. Use of placebo-relevant measures might have given additional insight into mechanisms of action for the treatment. Sixth, there were few very old participants (aged 80+), and we had limited information about frailty, which means that our findings most likely do not apply to frail older adults.

## Conclusions

We conclude that tailored therapist guided ICBT seems beneficial for older adults (65 years or older) with treatment effects maintained at one-year follow-up. Cognitive function needs to be studied further as a predictor, as it did not make an impact in this study. In addition to replicating the findings on tailored ICBT, future studies should examine more closely how cognitive function might influence treatment effects, and how ICBT can be further adapted to compensate for cognitive impairments.

## Electronic supplementary material

Below is the link to the electronic supplementary material.

**Additional file 1**: Content of the treatment modules.

**Additional file 2**: Outcomes at one-year follow-up for the treatment group, *n* = 36.

## Data Availability

The data analyzed during the study are available from the corresponding author on reasonable request.

## Abbreviations

CBT: Cognitive Behavioral Therapy
ICBT: Internet-delivered Cognitive Behavioral Therapy
GDS-15: Geriatric Depression Scale
BDI-II: Beck Depression Inventory
PHQ-9: Patient Health Questionnaire
RCT: Randomized Controlled Trial
M.I.N.I: Mini International Neuropsychiatric Interview
GAD-7: Generalized Anxiety Disorder scale
BAI: Beck Anxiety Inventory
QOLI: Quality of Life Inventory
CFQ: Cognitive Failures Questionnaire
WCST: Wisconsin Card Sorting Test-64
CI: Confidence Interval
M: Mean
SD: Standard deviation
