# Supplementary material for: Tailored internet-delivered cognitive behavior therapy for depression in older adults: a randomized controlled trial
Source: BMC Geriatr. 2024 Dec 10;24:998. doi: 10.1186/s12877-024-05597-8 (PMC11629493; doi:10.1186/s12877-024-05597-8)
Supplement: Supplementary file 1 — Additional file 1: Content of the treatment modules. [file 12877_2024_5597_MOESM1_ESM.docx]

**Additional file 1. Content of the treatment modules**

| Name of module | Standard module^a^ Yes/No | Content |
| --- | --- | --- |
| Introduction | Yes | Introduction of the principles and structure of treatment. Introduction to depression, associated symptoms, as well as to CBT in general. Exercises in the module are about describing how situations in life affect behavior, and what consequences this has for well-being (the ABC model), as well as designing treatment goals for the individual. |
| Behavioral activation 1 | Yes | An explanatory model of the principles of behavioral activation, and how activities in everyday life affect mental well-being. The focus of the module is on so-called “plus” activities, i.e., activities that in principle improve well-being. Exercises consist of mapping current activity patterns, identifying plus activities, and working out an activity plan in which the frequency of plus activities is increased. |
| Behavioral activation 2 | Yes | Extension of the previous behavior activation module. The focus of this module is on “minus” activities, i.e., activities that give rise to difficult feelings or are used to avoid difficult feelings, and also suggestions on how minus activities can be handled constructively. Exercises in the module are about dividing an activity into influenceable subcomponents, evaluating the activity plan from the previous module, designing a reward list, and designing a new activity plan for the coming week. |
| Acceptance | Yes | A review of different ways to deal with discomfort, as well as a description of the concept of acceptance in CBT. Exercises to map discomfort in everyday life, to explore previous coping strategies, and to explore one's own values in life. |
| Life goals and relapse prevention | Yes | A presentation of strategies for maintaining change, as well as dealing with setbacks. The module also emphasizes the importance of looking ahead with regard to values and goals in life. The exercises are about continuing with what has worked well in treatment so far, noting strategies for dealing with future setbacks, as well as ways to identify and formulate values and goals. |
| Sleep | No | A description of how sleep works, as well as the interaction with mental well-being. Description of factors that affect sleep, as well as strategies for a better sleep. Exercises to write a sleep diary, influence your own sleep factors, and to choose your own sleep strategies. |
| Anxiety and exposure | No | A description of what anxiety is and how it can be managed. Description of how short-term comforting thinking can paradoxically perpetuate long-term symptoms of anxiety, and how acceptance can be used to reduce the impact of anxiety on life. Practice on mapping and influencing the time spent on worrying thinking, as well as practice on mindfulness. |
| Emotion regulation | No | A review of what emotions are and how they can be identified and regulated. Description of a model where emotions are divided into 3 parts, namely 1) physiological reaction, 2) thought / interpretation and 3) behavior. Practice on identifying, mapping, and regulating emotions. |
| Loneliness | No | Description of what loneliness is and how it can be reduced. Exercises on mapping one's own interaction with other people, as well as identifying and applying strategies for connecting with other people. |
| Life review | No | To sum up life by exploring different time periods of life. Reflection on positive and negative events in different periods of life, and how these have been handled and contributed to lessons for the individual. |
| Pain | No | Description of what pain is, and how to develop an accepting approach to the pain. Distinction is made between short-term and long-term pain, and mindfulness is introduced into this context. Exercises to investigate past and alternative strategies for managing pain, as well as practice on mindfulness. |
| Relaxation | No | Description of relaxation in general, as well as different types of relaxation. Exercises with three different types of relaxation: muscle control, deep relaxation, and quick relaxation. |

Footnotes: ^a^ =Standard modules were given to all participants, while additional modules were added according to the tailoring of treatment (on basis of needs and preferences) to each unique participant.
