# Supplementary material for: Tailored internet-delivered cognitive behavior therapy for depression in older adults: a randomized controlled trial
Source: BMC Geriatr. 2024 Dec 10;24:998. doi: 10.1186/s12877-024-05597-8 (PMC11629493; doi:10.1186/s12877-024-05597-8)
Supplement: Supplementary file 2 — Additional file 2: Outcomes at one-year follow-up for the treatment group, n = 36. [file 12877_2024_5597_MOESM2_ESM.docx]

**Additional file 2. Outcomes at one-year follow-up for the treatment group, n=36**

| Measure | Pre  mean (SD) | I-year , follow-up,  mean (SD) | Within-group paired t-value; p-value | Within-group Cohens d (95%CI) |
| --- | --- | --- | --- | --- |
| GDS-15 | 8.6 (3.1) | 5.1 (3.7) | 5.41; <0.001 | 0.90 (0.51-1.29) |
| BDI-II | 22.2 (7.0) | 12.8 (8.3) | 7.12; <0.001 | 1.19 (0.75-1.61) |
| PHQ-9 | 12.4 (4.3) | 6.9 (4.7) | 6.94; <0.001 | 1.16 (0.73-1.58) |
| BAI | 14.5 (8.0) | 9.9 (7.1) | 3.87; <0.001 | 0.65 (0.28-1.00) |
| GAD-7 | 6.6 (3.8) | 4.3 (4.8) | 3.13; 0.003 | 0.52 (0.17-0.89) |
| QOLI | 0.5 (1.7) | 1.2 (1.6) | 2.54; 0.016 | 0.42 (0.08-0.76) |
| CFQ | 41.7 (13.2) | 37.2 (11.7) | 2.61; 0.013 | 0.44 (0.09-0.78) |

Note: GDS=Geriatric Depression Scale; BDI=Beck Depression Inventory; PHQ=Patient Health Questionnaire; BAI=Beck Anxiety Index; GAD=Generalized Anxiety Disorder; QOLI=Quality of Life Inventory; CFQ=Cognitive Failures Questionnaire.
